# Prevalence of positive chronic kidney Disease screening in professional male long haul drivers at risk of cardiovascular Disease in Lagos, Nigeria: a cross-section study

**DOI:** 10.1186/s12889-019-7328-6

**Published:** 2019-08-01

**Authors:** Casmir E. Amadi, Amam C. Mbakwem, Oyewole A. Kushimo, Jayne N. Ajuluchukwu, Michael Akinkunmi

**Affiliations:** 1 Department of Medicine, College of Medicine, University of Lagos, Lagos, Nigeria; 2 Department of Medicine, Lagos University Teaching Hospital, Lagos, Nigeria

**Keywords:** Cardiovascular disease, Chronic kidney Disease, eGFR, Risk factors, Professional drivers

## Abstract

**Background:**

Professional drivers are known to be at high risk for cardiovascular disease (CVD) on account of the higher prevalence co-occurring risk factors they harbour. Chronic kidney disease (CKD) and CVD share similar risk factors. Both impact each other adversely. The renal profile of professional drivers in Nigeria is not well characterised. We decided to study the prevalence of positive CKD screening amongst professional male long distance drivers in Lagos, Southwest Nigeria so as to quantify the burden and its predictors.

**Methods:**

Two hundred and ninety-three drivers were recruited. Details of their socio-demographic characteristics were obtained. Their anthropometric indices, blood pressure, fasting plasma blood glucose and lipid profile were measured. Serum creatinine was measured and estimated glomerular filtration rate, eGFR, was calculated with Chronic Kidney Disease Epidemiology Collaboration (CKD-EPI) equation. Urinary Albumin Creatinine Ratio (UACR) was determined. A 10 year CVD risk of the subjects was calculated with the Framingham Risk Score (FRS).

**Results:**

Mean age of the study population was 44.8 + 9.7 years. The prevalence of alcohol use and smoking were 71.0 and 19.5% respectively. One hundred and twenty-one (62.8%) of the subjects were either overweight or obese while 70(24.1%) had abdominal obesity. The prevalence of hypertension and diabetes were 39.7 and 13.9% respectively. Prevalence of CKD by eGFR< 60 ml/min/1.73m^2^ or UACR > 30 mg/g was 51.7% (95% CI; 46.0–57.5). The odds for CKD increased with lower HDL-c levels; OR 3.5 (95% CI, 1.1–11.2; *p* = 0.03) and longer duration of professional driving > 20 years; OR 2.4(95% CI, 1.5–4.0).

**Conclusion:**

Professional male long distance drivers in addition to having very high prevalence of clustering of both CVD and CKD risk factors have a significant burden of asymptomatic CKD. UACR appears to be an earlier marker of CKD in this population. Health awareness promotion and aggressive risk factor reduction are advocated as ways to reduce this burden.

## Introduction

Chronic kidney disease (CKD) is an important cause of global morbidity and mortality with an estimated prevalence of 14.3% with the majority in advanced stages [1]. The burden is on the increase and it is driven largely by the rising prevalence of CKD risk factors such as hypertension, diabetes, smoking, obesity and dyslipidaemia [2]. Low-Middle Income Countries (LMICs) account for a huge fraction of this burden due to epidemiologic transition [2].

CKD and Cardiovascular disease (CVD) are intimately related. In addition to sharing common risk factors, CKD heightens CVD risk and is an independent predictor of CVD events [3, 4]. Both are asymptomatic in the early stages. CVD is the commonest cause of death in individuals with both early and late stages of CKD [3, 5]. Both also exert high economic cost on healthcare systems [6, 7]. Opportunistic screening for risk factors for both conditions and their optimal control remain cost-effective strategies to reduce their burgeoning burden [8, 9].

CKD prevalence is driven globally by high prevalence of its risk factors which often times are poorly controlled [10, 11]. A recent multinational study that included 12 LMICs reported CKD prevalence of 14.3% in the general population and 36.1% in the high risk population [1]. Low awareness of the disease especially in its early asymptomatic phase is said to account for the rising burden also [1]. This low awareness leads to a large number of individuals progressing to End Stage Renal Disease (ESRD).

In Nigeria the prevalence of CKD varies from 1.8 to 43.5% depending on the studied population characteristics and methods of estimating glomerular filtration rate [1, 12–15]. These studies also identified hypertension, dyslipidaemia, diabetes, overweight/obesity and age as risk factors for CKD, which are also CVD risk factors.

Professional drivers are known to have high risk of CVD more than the general population due to the plethora of multiple and co-occurring risk factors for CVD they harbour [16–18]. Most times these risk factors are detected only through opportunistic screening, with individuals usually unaware of them [18]. In Nigeria studies have reported higher prevalence of CVD risk factors such as hypertension, abnormal glucose profile, obesity, dyslipidaemia, smoking, alcohol consumption and physical inactivity in this group more than in the general population [18–20]. The guidelines recommend that CKD screening be carried out in individuals with risk factors for CKD and CVD [21, 22]. However there are no studies on the prevalence of asymptomatic CKD in this economically vibrant but vulnerable segment of the population. Since CVD risk factors are known to be higher in them than in the general population, we hypothesised that asymptomatic CKD would also be higher in them. We therefore decided to study the prevalence of positive CKD screening in professional male long distance drivers with high prevalence of clustering of CVD risk factors in Lagos, South West Nigeria.

## Methods

This was a cross-sectional study involving male long distance drivers in major Motor Parks in in Lagos. The Parks were selected based on their size and the routes they serve. Long distance driving was defined as a distance of 300 km radius from the terminal of departure “(https://www.aa.co.za/insights/long-distance-driving%20tips)”.

Details of sample size calculation, recruitment and selection of the participants were described in our previous publication [18].

### Ethics

Ethical Approval (No: ADM/DCST/HREC/APP359) for the study was obtained from the Health Research Ethics Committee of the Lagos University Teaching Hospital. All consenting drivers signed a written consent form.

### Data collection

The subjects were met in groups, received a health talk on the benefits of healthy living and also briefed on the rationale and importance of the research. They were also asked to observe an overnight fast on the agreed day of the medical screening. A structured questionnaire administered by trained interviewers was used to obtain their socio-demographic data and relevant medical history. Linguistically-appropriate translators were used to assist subjects who didn’t understand English language to complete the questionnaire.

### Smoking and alcohol habits

Smoking status was elicited from the subjects. Smokers were those who smoked at the time of the study, ex-smokers, those who quit smoking at least twelve months before the commencement of the study, and non-smokers, those who had not smoked all their lives. Alcohol “users” were those who consumed alcoholic beverages at the time of the study while teetotallers were abstainers.

### Physical measurements

The body weight (in kilograms) of the subjects was measured with an Omron HN289(Osaka Japan) digital weighing scale, placed on a firm and even surface with subjects wearing light clothing and no footwear or cap. Measurements were taken to the nearest 0.5 kg ensuring that the scale was at the zero mark always. Their heights(in centimeters) were measured with a Seca model 216 (GmbH, Hamburg, Germany) Stadiometer ensuring the subjects were standing erect, back against the height meter rule and occiput and heels making contact with the height meter rule. Body Mass Index (BMI) was calculated as weight in kilograms divided by height^2^ in meters [23]. Overweight was defined as BMI > 25 kg/m^2^ while obesity as BMI ≥ 30 kg/m^2^. Waist circumference was measured with an inextensible, inelastic 1-cm wide tape snug around the body at the level of the midpoint between the lower margin of the last palpable rib and the top of the anterior iliac crest. Measurements were taken at the end of normal respiration and ≥ 102 cm was regarded as abdominal obesity [24]. The blood pressure (BP) was measured with Omron-branded digital sphygmomanometer (HEM7233 Osaka, Japan) after 5 min of rest with the subject seated comfortably, feet on the floor, and arm at the level of the heart and free of any constricting clothing. Initial measurements were on both arms and the arm with the higher value was used in subsequent measurements. Three BP readings were taken at 2–3 min intervals. The average of three readings was taken for analysis. Hypertension was defined as BP ≥ 140/90 mmHg, self-volunteered history of hypertension and/or use of anti-hypertensives.

### Biological specimens

Ten millilitres of venous blood taken from a visible peripheral vein appropriate was put vacutainer specimen bottles for fasting plasma glucose, fasting lipid profile, serum creatinine and albumin. Samples were analysed with a clinical chemistry autoanalyzer using standard reagents/kits from Randox Laboratories “(www.randox.com/diagnostic-reagents/)”. Subjects with a fasting plasma glucose value of ≥7.0 mmol/l, self-volunteered history of diabetes and or use of insulin/ oral hypoglycemic agents were regarded as diabetic while fasting plasma glucose level between 5.5 and 6.9 mmol/l was regarded as impaired fasting glucose [25]. Abnormal lipid profile was defined as: total cholesterol > 6.6 mmol/l, HDL-c < 1.04 mmol/l, and LDL-c > 4.1 mmol/l and triglyceride > 1.7 mmol/l [26]. Atherogenic dyslipidaemia was defined by the Castelli Index as Tc/HDL-c > 3.4 [27]. Serum creatinine was measured by the modified Jaffe method using the Beckman creatinine analyser. The estimated glomerular filtration rate, eGFR in ml/min per 1.73m^2^, was estimated by the Chronic Kidney Disease-Epidemiology Collaboration, CKD-EPI equation [28] based on serum creatinine, age, sex and a two-level racial variable (African-American or Non-African American). The CKD-EPI equation was used in preference to the MDRD equation because the former underestimates eGFR at higher values [29]. Spot urine samples were collected in sterile 30 ml universal bottles for estimation of albumin creatinine ratio. Albumin was quantified by immunoturbimetric methods. Subjects were then classified according to the Kidney Disease: Improving Global Outcomes (KDIGO) 2012 CKD classification. We defined significant positive CKD screening as eGFR as (< 60 ml/min per 1.73m^2^) and or UACR ≥30 mg/g [22].

### Framingham risk score

A 10 year CVD risk of the subjects was calculated using the Framingham Risk Score. This risk calculator is a downloadable app on smart phones and incorporates 7 variables namely age, sex, smoking status, systolic BP, total cholesterol, HDL-c and treatment of hypertension with medications. A score > 20% is classified as high risk.

### Statistical analysis

Data entry and analysis were done with the Statistical Package for the Social Sciences 22.0 version (SPSS, Inc., Chicago IL USA). Numerical data was presented as mean and standard deviation or as median and interquartile range for skewed variables. Categorical data were expressed as proportions. Associations between cardiovascular disease risk factors and positive CKD screening were sought using the chi -square test with a level of significance set at *p* < 0.05. Corresponding odds ratios were calculated with confidence intervals set at 95%. Multivariate analysis was done using a forward stepwise binary logistic regression in order to assess independent predictors of renal dysfunction. We included predictor variables with associations at a significance level of *p* < 0.1 to accommodate for important CVD risk factors.

## Results

A total of 308 drivers were recruited for the study. Fifteen of them were excluded due to incomplete data. Thus 293 were used for data analysis.

### Socio-demographic characteristics

The mean age of the subjects was 44.8 ± 9.7 years; 139(47.4%) of them aged between 45 and 65 years. The rest of their socio-demographic characteristics are shown in Table 1. Fifty-seven of the subjects (19.5%) were active smokers while 208 (71.1%) of them used alcohol (Table 1).

**Table 1:** Socio-Demographic Characteristics of the Subjects

Parameters	Mean + (SD)	N (%)
Age (years),=293	44.8 + 9.7	
*25–44*		147(50.2)
*45–64*		139(47.4)
*>65*		7(2.4)
Education Attainment,=293		
*Primary*		77 (26.3)
*Secondary*		177 (60.4)
*Tertiary*		37 (12.6)
Marital Status,=293		
*Married*		265 (90.4)
*Single*		22 (7.5)
*Widowed*		3 (1.0)
*Divorced*		3(1.0)
Number of Years as a Pro driver	20.0 + 10.4	
Number of Hours		
Driven per week	41.9 + 28.7	
Smoking Pattern,=293		
*Active Smokers*		57(19.5)
*Non-Smokers*		217(74.1)
*Ex-smokers*		19(6.5)
Alcohol use,=293		
*User*		208(71.0)
*Teetotalers*		85(29.0)

### Measures of obesity

The mean BMI of the subjects was 27.2 ± 9.6 kg/m^2^. The prevalence of overweight and obesity 121(41.7%) and 61(21.1%) were respectively. The mean waist circumference of the study population was 94.9 + 11.9 cm, with 24.1% of them having abdominal obesity (WC ≥ 102 cm).

### Blood pressure profile

The mean Systolic Blood Pressure (SPB) and Diastolic Blood Pressure (DBP) of the subjects were 136.3 + 20.9 mmHg and 83.2 + 13.6 mmHg respectively. One hundred and sixteen (39.7%) cases of hypertension were identified with 75.9% of them were being previously undiagnosed. Twenty-eight (24.1%) of them were previously known hypertensives (Table 2).

**Table 2:** Cardio-renal risk factors of the Subjects

Parameter	Mean ± SD	N (%)
BMI (kg/m^2^)	*27.2* *+* *9.6*	
Waist Circumference(cm)	*96.4* *+* *0.9*	
*Proportion ≥ 102 cm, n = 291*		*70(24.1)*
Blood Pressure		
*SBP(mmHg)*	*136.3 ± 20.9*	
*DBP(mmHg)*	*82.2 ± 13.6*	
*Prevalence of Hypertension, n = 292*		*116(39.6)*
Blood Glucose*, n = 288*		
*FBG(mmol/l)*	*6.01 ± 2.2*	
*Prevalence of Impaired Fasting Glucose*		*90(31.3)*
*Prevalence of Diabetes*		*40(13.9)*
Lipid Profile		
*T*_*C*_*(mmol/l)*	*5.66 ± 0.86*	
*LDL-c(mmol/l)*	*3.53 ± 0.87*	
*HDL-c(mmol/l)*	*1.49 ± 0.40*	
*T*_*G*_*(mmol/l)*	*1.39 ± 0.72*	
*T*_*C*_*/HDL-c*	*3.8 ± 1.9*	
Abnormal Profile		
*Elevated T*_*C*_*, n = 291*		*81(27.8)*
*Elevated LDL-c, n = 293*		*72(24.6)*
*Low HDL-c, n = 292*		*19(6.5)*
*Elevated T*_*G*_*, n = 293*		*72(24.6)*
*Elevated T*_*c*_*/HDL-c, n = 290*		*96(33.1)*
*Renal Indices*		
*Serum Creatinine (*μmmol*/l)*	*105.6* ±29.9	
*UACR (mg/g)*	*28.4(16.1–57.9)*	
*eGFR*	*96.0 ± 57.4*	
*Framingham Risk Category, n = 291*		
*Low*		*186 (63.6)*
*Intermediate*		*76 (25.8)*
*High*		*31 (10.7)*

*BMI* Body Mass Index, *WC* Waist Circumference, *BP* Blood Pressure, *FBG* Fasting Blood Glucose, *T*_*C*_ Total Cholesterol, *LDL-c* Low Density Lipoprotein cholesterol, *HDL-c* High Density Lipoprotein cholesterol, *T*_*G*_ Triglyceride, *UACR* Urine Albumin Excretion Ratio

### Glycaemic profile

The mean FBG of the study population was 6.0 ± 2.2 mmol/l. The prevalence of diabetes was 13.9%. Seven (2.4%) of the subjects were previously known diabetics. Ninety (31.3%) of the subjects had impaired fasting glucose.

### Lipid profile

The mean Tc of the study population was 5.7 ± 0.86 mmol/l. The overall lipid profile is presented in Table 2. Prevalence of dyslipidaemia was 56.3% while atherogenic dyslipidaemia i.e. elevated Tc/HDL-c was seen in 96(33.1%) of the subjects (Table 2).

### Renal function indices of the subjects

The mean serum creatinine of the subjects was 105.6 ± 29.9 μmol/l while the median value urinary albumin creatinine excretion ratio (UACR) was 28.4 (IQR;16.1–57.9)mg/g. The mean eGFR was 96.02 ± 57.4 ml/min (Table 2).

### Framingham risk score

The mean Framingham Risk Score (FRS) of the subjects was 9.8 ± 8.6%. Majority (63.6%) were low risk while 25.8 and 10.7% of the subjects were in the intermediate and high risk categories respectively (Table 2).

### Prevalence of CKD

The prevalence of positive CKD screening defined as eGFR < 60 ml/min or UACR > 30 mg/g was 51.7% (95% CI; 46.0–57.5). Prevalence of CKD using eGFR < 60 ml/min alone was 8.2% (95% CI, 5.1–11.3) and 47.2% (95% CI, 41.5–53.0) using UACR 30 mg/g alone. Table 3 shows the prevalence of CKD of the subjects according to their eGFR and UACR categories. Prevalence of KDIGO CKD stages were: stage 1; 62(21.4%), stage 2; 65(22.4%), stage 3a; 21(7.2%), stage 3b; 1(0.3%) and stage 4; 1(0.3). Only one subject was in stage 3b and hence stage 3b was merged with Stage 3a to yield stage 3 only (Table 4). Table 4 also shows the distribution of their socio-demographic and CKD/CVD risk factors of the subjects according to their KIDGO CKD staging.

**Table 3:** Prevalence of CKD in the subjects according to the KIDGO CKD Staging

eGFR categories KDIGO		KDIGO UACR groups			Total n (%)
		A1:< 30 mg/g n(%)	A2:30-300mg/g n(%)	A3:> 300 mg/g n(%)	
	G1(> 90)	58(20.0)	57(19.7%)	3(1.0)	118(40.7)
	G2(60–89)	82(28.3)	62(21.4)	4(1.4)	148(51.0)
	G3a(45–59)	12(4.1)	10(3.4)	0(0.0)	22(7.6)
	G3b(30–44)	1(0.3)	0(0.0)	0(0.0)	1(0.3)
	G4(15–29)	0(0.0)	1(0.3)	0(0.0)	1(0.3)
Total		153(52.8)	130 (44.8)	7 (2.4)	290 (100.0)

*eGFR* Estimated Glomerular filtration Rate, *UACR* Urine Albumin Excretion Ratio, *KIDGO* Kidney Disease: Improving Global Outcomes, *CKD* Chronic Kidney Disease

**Table 4:** Distribution of socio-demographic characteristics and risk factors of subjects according to their KIDGO CKD Staging

Parameter	No CKD	Stage 1 (eGFR > 90; uacr > 30)	Stage 2 (eGFR 60–89; uacr > 30)	Stage 3 (eGFR 30–59)	Stage 4 (eGFR 15–29)
Age					
*>50 years n(%);(n = 97)*	39(40.2)	20(20.6)	26(26.8)	11(11.3)	1(1.0)
*<50 years n(%);(n = 193)*	101(52.3)	42(21.8)	39(20.2)	11 (10.9)	0(0)
Education					
*Primary n(%);(n = 77)*	25(32.5)	24(31.2)	22(28.6)	5(6.5)	1(1.3)
*Secondary n(%);(n = 175)*	99(56.6)	29(16.6)	34(19.4)	13(7.4)	0(0)
*Tertiary n(%);(n = 37)*	16(43.2)	8(21.6)	9(24.3)	4(10.8)	0(0)
*Postgraduate n(%);(n = 1)*	0(0)	1(100)’	0(0)	0(0)	0(0)
Marital Status					
*Single n(%);(n = 22)*	12(54.5)	2(9.1)	8(36.2)	0(0)	0(0)
*Married n(%);(n = 262)*	125(47.7)	60(22.9)	56(21.4)	21(8.0)	0(0)
*Divorced n(%);(n = 2)*	1(50.0)	0(0)	0(0)	1(50.0)	0(0)
*Widowed n(%);(n = 3)*	1(33.3)	0(0)	1(33.3)	1(33.3)	1(33.3)
Years of Professional driving					
*≥20 years n(%);(n = 142)*	56(39.4)	31(21.8)	37(26.1)	17(12.0)	1(0.7)
*<20 years n(%);(n = 142)*	83(58.5)	31(21.8)	25(17.6)	3(2.1)	0(0)
Driving hrs / week					
*≥36 n(%);(n = 147)*	80(54.4)	23(15.6)	27(18.4)	17(11.6)	0(0)
*<36 n(%);(n = 142)*	59(41.5)	39(27.5)	38(26.8)	5(3.5)	1(0.7)
Smoking n(%);(*n* = 56)	29(51.8)	10(17.9)	14(25.0)	3(5.4)	0(0)
Alcohol n(%);(*n* = 205)	100(48.8)	41(20)	44(21.5)	20(9.8)	0(0)
WC					
*≥102 cm n(%);(n = 68)*	24(35.3)	20(29.4)	15(22.1)	8(11.8)	1(1.5)
BMI					
*Overweight/Obesity n(%);(n = 180)*	86(47.8)	37(20.6)	38(21.1)	18(10.0)	1(0.6)
Hypertension n(%); (*n* = 114)	48(42.1)	25(21.9)	23(20.2)	17(14.9)	1(0.9)
Abnormal Glucose Profile n(%)					
*IFG (n = 90)*	50(55.6)	11(12.2)	19(21.1)	10 (11.1)	0(0)
*Diabetes (n = 39)*	17(43.6)	9(23.1)	10(25.6)	3(7.3)	0(0)
Abnormal Lipid Profile n(%)					
*Elevated T*_*C*_ *(n = 79)*	36(45.6)	20(25.3)	21(26.6)	2(2.5)	0(0)
*Elevated LDL (n=71)*	35(49.3)	13(18.2)	20(28.2)	3(4.2)	0(0)
*Low HDL (n=19)*	5(26.3)	0(0)	10(52.6)	4(21.1)	0(0)
*Elevated T*_*G*_ *(n = 69)*	34(49.3)	15(21.7)	16(23.2)	4(5.8)	0(0)
*Elevated T*_*C*_*/HDL (n = 95)*	39(41.1)	33(34.7)	16(16.8)	6(6.3)	1(1.1)
FRSn(%)					
*Low (n = 183)*	96(52.5)	40(21.9)	38(20.8)	9(4.9)	0(0)
*Intermediate (n = 75)*	30(40.0)	14(18.7)	21(28.0)	10(13.3)	0(0)
*High (n = 30)*	13(43.3)	7(23.3)	6(20.0)	3(10.0)	1(3.3)
*BMI(Mean ± SD)*	26.6 ± 4.3	26.8 ± 4.7	26.3 ± 4.3	27.8 ± 4.2	32.7*

*BMI* Body Mass Index, *WC* Waist Circumference, *BP* Blood Pressure, *FBG* Fasting Blood Glucose, *T*_*C*_ Total Cholesterol, *LDL-c* Low Density Lipoprotein cholesterol, *HDL-c* High Density Lipoprotein cholesterol, *T*_*G*_ Triglyceride, *FRS* Framingham Risk Score, *eGFR* Estimated Glomerular filtration Rate, *UACR* Urine Albumin Excretion Ratio, *KIDGO* Kidney Disease: Improving Global Outcomes, *CKD* Chronic Kidney Disease

*single value for one subject

### CKD stages and CVD risk

For subjects with CKD stage 1, seven (11.5%) were in high risk Framingham Risk Score (FRS) category, while for stages 2, 3 and six (9.2%), three (14.3%), and one (100%) were in high FRS category respectively.

### Association CVD/CKD risk factors with positive CKD screening

The association between CKD and CVD risk factors and some characteristics of the subjects in univariate analysis is shown in Table 5. Only WC, number of driving hours per week ≥36 h, years of professional driving ≥20 years and low HDL-c were significantly associated CKD. In logistic regression analysis the odds of CKD increased with low HDL-c and long years of professional; 3.5(95% CI; 1.1–11.2; *p* = 0.03) and 2.5(95% CI; 1.5–4.0; *p* < 0.001) respectively (Table 6). Number of driving hours ≥36 h per week had the lowest odds for CKD in this model; OR 0.5(95% CI, 0.34–0.89; *p* = 0.002).

**Table 5:** Univariate Association between CVD Risk factors and CKD/Albuminuria

Parameter	CKD positive screen (eGFR < 60 ml/m^2^ or uacr > 30 mg/g^)^				Albuminuria positive screen (uacr > 30 mg/g)			
	N (%)	*p* value^b^	OR^a^	95% CI	N (%)	*p* value^b^	OR^a^	95% CI
Hypertension		0.10	1.49	0.93–2.40		0.82	1.06	0.66–1.70
Present	66 (57.9)				55 (48.2)			
Absent(ref)	84 (48.0)				82 (46.1)			
Abnormal glucose		0.39	0.89	0.51–1.30		0.82	1.06	0.64–1.77
Present	62(48.1)				39 (47.6)			
Absent(ref)	83(53.2)				94 (46.1)			
HDL-c		0.046	2.80	1.00–7.99		0.14	2.02	0.77–5.23
Low	14 (73.7)				12 (63.2)			
Normal(ref)	135 (50.0)				124(45.9)			
LDL-c		0.84	0.95	0.56–1.62		0.69	1.12	0.66–1.90
Elevated	36 (50.7)				35 (49.3)			
Normal(ref)	114 (52.1)				102(45.9)			
Driving hrs/wk		0.03	0.60	037–0.95		0.001	0.46	0.29–0.74
≥36	67 (45.6)				56 (38.1)			
<36(ref)	83 (58.5)				81 (57.0)			
Prof driving yrs.		0.001	2.16	1.35–3.47		0.024	1.72	1.07–1.75
≥20	86 (60.6)				76 (53.5)			
<20(ref)	59 (41.5)				57 (40.1)			
Smoking		0.56	0.84	0.47–1.51		0.67	0.88	0.49–1.58
Yes	27 (48.2)				25 (44.6)			
No(ref)	123 (52.6)				112(47.9)			
Waist circumference		0.02	1.97	1.12–3.46		0.03	1.81	1.04–3.14
≥102 cm	44 (64.7)				40 (58.8)			
<102(ref)	106 (48.2)				97 (44.1)			
BMI		0.77	1.07	0.67–1.73		0.87	0.96	0.60–1.55
Overweight/obese	94 (52.2)				84 (46.7)			
Normal(ref)	54 (50.5)				51 (47.7)			
Age		0.051	2.64	1.00–2.68		0.12	1.47	0.90–2.40
≥50	58 (59.8)				52 (53.6)			
<50(ref)	92 (47.7)				85 (44.0)			
Alcohol Use		0.79	0.93	0.56–1.55		0.46	0.82	0.50–1.37
Yes	105 (51.2)				94 (45.9)			
No(ref)	45 (52.9)				43 (50.6)			
Framingham Risk score		0.06	1.69	0.98–2,59		0.18	1.39	0.86–2.24
>10%	62(59.0)				55 (52.4)			
<10%	87(47.5)				81 (44.3)			

^a^Unadjusted odds ratios; ^b^ Chi-Square, *eGFR* Estimated Glomerular filtration Rate, *UACR* Urine Albumin Excretion Ratio, *KIDGO* Kidney Disease: Improving Global Outcomes, *CKD* Chronic Kidney Disease, *BMI* Body Mass Index, *LDL-c* Low Density Lipoprotein Cholesterol, *HDL-c* High Density Lipoprotein Cholesterol

**Table 6:** Logistic Regression for Predictors of CKD (eGFR< 60 ml/min or UACR> 30 mg/g) and albuminuria (UACR > 30 mg/g)

	eGFR<60 ml/min or UACR > 30 mg/g^a^			UACR > 30 mg/g^b^		
Variable	Adjusted OR	95%CI	*P*-value	Adjusted OR	95%CI	*P*-value
Professional Driving ≥20 years	2.4	1.5–4.0	< 0.001	1.7	1.1–2.8	0.02
Low HDL-C	3.5	1.1–11.2	0.03	–	–	–
Driving Hours per week≥36 h	0.5	0.3–0.9	0.01	0.4	0.3–0.7	0.001

^*a*^ *Independent variables excluded from the final model by forward stepwise method include hypertension, waist circumference, age and Framingham Risk Score*

^*b*^ *Independent variable excluded from the final model by forward stepwise method was waist circumference*

## Discussion

This study, first of its kind (to the knowledge of the authors), assessed the prevalence of positive single CKD screening in male long distance bus drivers known to have high prevalence of CVD risk factors. The prevalence of positive CKD screening, eGFR< 60 ml/min per 1.73m^2^ and UACR > 30 g/mg was 51.7%. Studies carried out in the general population across Nigeria have reported prevalence rates ranging from 1.8 to 43.5% [1, 12–15]. There are no similar local or sub-Saharan Africa studies for comparison. This wide variation in prevalence may be largely methodological; peculiarities of the study population and use of the Cockcroft-Gault formula or the MDRD equation which overestimate eGFR [28, 29].The higher prevalence reported from our study might be due the high CVD risk of our study population since the prevalence of CKD in published literature is reported to be higher in high risk populations such as ours [1] and the fact that we used the combined criteria of eGFR< 60 ml/min and UACR > 30 mg/g according to the KIDGO guidelines [22]. A study of CKD prevalence using the MDRD equation in taxi drivers in Iran, with mean age similar to our study, reported a lower prevalence rate of 6.4% [30]. Our study involved long distance drivers with multiple and higher prevalence of CKD risk factors, longer duration of professional driving and longer weekly hours of driving and these might account for our higher prevalence. The prevalence of CKD with urinary albumin excretion ratio (UACR) > 30 g/mg from our study was 47.2%. This is much higher than values obtained from both local and international studies done in the general population [1, 12, 13, 31–33]. However there are no known studies in professional drivers for comparison. Probably a higher UACR may be a peculiar feature in this group. UACR is known to be an earlier marker of CKD than eGFR and also of endothelial dysfunction, the pathologic surrogate of atherosclerotic CVD [22]. The ability of UACR to detect sub-clinical CKD earlier than eGFR might be due to the fact that all the eGFR prediction equations are based on serum creatinine, which is influenced by diets, muscle mass, intrinsic creatinine glomerular filtration, tubular secretion, colorimetric interference and day-to-day assay variability [34]. Thus early detection of high UACR is a pointer to possible kidney damage which should instruct aggressive search for and treatment of modifiable risk factors and use of nephron-protective drugs like Angiotensin Enzyme Converting Inhibitors (ACEI) and Angiotensin Receptor Blockers (ARB) known to slow down CKD progression [21].

Several risk factors have been causally associated with CKD. These include advancing age, hypertension, obesity, abnormal glucose profile, dyslipidaemia, and smoking, which are also traditional risk factors for CVD [35, 36]. In our study age 50 years, waist circumference ≥ 102 cm, low HDL-c, number of driving hours per week ≥36 h and number of years of professional driving ≥20 years were found to be significantly associated with risk of CKD in univariate analysis. However in multivariate logistic regression model only low HDL-c, number of driving hours per week ≥36 h and number of years of professional driving ≥20 years were found to be significant predictors of CKD. Number of hours of driving per week ≥36 h had lower odds for CKD.

Age is a determinant of renal function. GFR is believed to decline by 1 mL/ml/1.72m^2^ per year after the age of 30 years in healthy persons [30]. In the Chronic Renal Disease In Turkey (CREDIT) study the odds ratios of CKD ranged from 1.45 to 2.18 for every 10-year increase in age among subjects older than 30 years of age in Turkey [37]. Most studies reported age > 50 years as a predictor of CKD [1, 12–14, 31–33]. In our study with a population mean age 44.8 years, 11.6% of those above 45 years had CKD while 59.8% of those above 50 years had CKD, corroborating reports from the above studies. Mahdavi-Mazdeh et al. [30] reported a CKD prevalence of 20.3% among taxi drivers above 60 years in Iran. The importance of advanced age as an important risk factor for CKD is further buttressed by the guidelines [21, 22].

Hypertension is an established risk factor for both CKD and CVD. CKD and hypertension are intimately related with an overlapping and intertwined cause and effect relationship. Sustained and uncontrolled hypertension is associated with decline in kidney function while declining kidney function leads to increase in BP [38]. Hypertension is the commonest risk factor associated with CKD. In the Chronic Renal Insufficiency Cohort (CRIC), which comprised 3612 adults with CKD (majority in moderate stage), the prevalence of self-reported hypertension was 86% compared with 29% in the general population [39]. In our study 39.7% of the subjects were hypertensive compared to the 28.9% prevalence in the general population [40]. Of those with hypertension 57.8% of them had CKD, making it one of the commonest associates of CKD in univariate model. Declining kidney function due to hypertension invariably leads to ESRD. To stem the rising burden of ESRD aggressive pharmacological treatment of hypertension to goal of < 130/80 mmHg is a guideline directed preventive measure [41, 42].

Obesity shares an intimate relation with and the development and the progression of CKD. While obesity is associated with the presence and development of proteinuria in individuals without kidney disease, independent of traditional CVD risk factors such as hypertension and diabetes, it is a risk factor for hypertension and diabetes, both also established risk factors for CKD [43, 44]. The prevalence of overweight/obesity in our study was 62.8%, much higher than rates reported in the general population [45]. Of this, 52.2% had CKD. Other studies also reported high prevalence of obesity in CKD cohorts [46, 47]. Waist circumference rather than BMI is said to be a better measure of obesity and correlates more accurately with CKD risk and CVD outcomes [48]. In our study waist circumference rather BMI was significantly associated with CKD in univariate analysis, with 64.7% of those with abdominal obesity having CKD. The exact mechanism of obesity induced CKD is not well known but may include inflammation, oxidative stress, abnormal lipid metabolism, activation of the Renin Angiotensin Aldosterone System (RAAS) and hyperinsulinaemia [49, 50]. In overweight and obese individuals weight reduction via caloric restriction and increased physical activity has been shown to reduce incident CKD by 30% [51]. In the Ramipril Efficiency In Nephropathy (REIN) study, the reno-protective effect of ramipril, an ACEI, was maximal in obese proteinuric non-diabetic subjects [52]. Thus lifestyle modification and use of ACEI when indicated can reduce the risk of incident CKD and its progression in obese individuals.

Perturbations in lipid metabolism have been associated with the onset and progression of CKD. The common lipid abnormalities in CKD are elevated triglyceride and low high density lipoprotein cholesterol (HDL-c) levels [53, 54]. Studies have shown that low HDL-c is a predictor of incident CKD and its progression [55, 56]. Reduced plasma levels of Apo-lipoproteins Apo A-I and Apo A-II are thought to underpin the aetiopathogenesis of low HDL cholesterol (HDL-c) levels seen in CKD [54]. In addition the quality of the HDL-c in CKD is poor, further worsening the dyslipidaemic milieu [56]. In our study 73.3% of subjects with low HDL-c had CKD and low HDL-c was also the only dyslipidaemia that predicted CKD in regression analysis. Low HDL-c in addition is a risk factor for CVD and may worsen outcomes in CKD by increasing CVD risk [53].

Long duration of professional driving ≥20 years and number of driving hours ≥36 h per week were others predictors of CKD from our study. They can be likened to surrogates of sedentariness and can contribute to abdominal obesity, which was highly prevalent in our study subjects and which was also associated with risk of CKD in univariate analysis. Both also are known predictors of metabolic syndrome in professional drivers of which abdominal obesity and low HDL-c are components of [57, 58]. Counter-intuitively long hours of driving per week had lower odds for CKD unlike longer years of duration of driving. Whether this represents a protective effect against CKD is unknown. It might be that age could be a potential confounder in this scenario, with younger individuals driving for ≥36 h/week. Further investigations in this area might yield more plausible explanations.

Smoking, use of alcohol and abnormal glucose profile, though known risk factors for CVD, were not significant predictors of CKD in our study. This might be methodological. Duration and quality of control of diabetes and duration and quantum of smoking are associated with risk of CKD [59, 60]. For smoking, studies have demonstrated that CKD risk is associated with cumulative lifetime cigarette exposure of > 25 pack years [60]. Our study did not assess both the duration of diabetes, quality of diabetic control with glycosylated haemoglobin and quantum of smoking of the subjects.

Lower stages of eGFR and higher levels of albuminuria are regarded as high CVD risk state [1, 21]. Our study showed that a 10 year risk of CVD assessed by the Framingham Risk Score increased with decreasing eGFR although the risk calculus was highest for the intermediate FRS category. However FRS category was not significantly associated with risk of CKD in univariate analysis.

Our study has some limitations. First this opportunistic screening tests involving creatinine and albumin were done only once with no obligatory 3 months re-screening to ascertain the persistence or otherwise of CKD as recommended by guidelines [21, 22]. Hence false positive results could have added to the prevalence CKD in some of the subjects with transient changes in these parameters. Although this one time screening has been used in population based studies in published literature, at the individual level obligatory re-screening to confirm initial abnormalities is always advised [21, 22]. To better define the management strategy at population and individual levels, results obtained during screening, in which one calculation for eGFR and one evaluation of UACR was done, could be better described as the prevalence of a positive screening test for chronic kidney disease, to which we refer in our study, while using the generally accepted term chronic kidney disease.

Secondly the CKD-EPI formula is said not to perform well in estimating GFR in sub-Saharan Africans with a tendency towards over-estimation [61, 62]. Thus values of eGFR reported in our subjects may be spuriously higher.

Finally the CKD-EPI formula requires the use of standardized serum creatinine values with a sophisticated costly reference standard. We did not do this due to its prohibitive cost. Our creatinine results were only standardized according to the reagent manufacturer’s guideline via Jaffe reaction. This might have affected the performance of the CKD-EPI formula in estimating GFR.

## Conclusion

Our study was able to document a higher prevalence of cluster of CKD risk factors as well as higher prevalence of CKD in professional male long haul drivers than in the general population. From Public health perspective it is pertinent to note that most of the risk factors they harbour are either preventable or modifiable through lifestyle adjustments and pharmacotherapy. Contributing to their risk also is their inverse social gradient which affects their access to healthcare and adherence to medical intervention. Primary prevention strategies likely to have large “effect size” in this economically important population include public awareness campaign on the importance of healthy lifestyles, banning of smoking and selling of alcohol beverages in motor parks, compulsory pre-employment and annual in-service health screening, defined maximum number of years of professional driving, occupational rehabilitation for drivers exceeding this limit and establishment of health posts in the motor parks to diagnose, treat and monitor risk factor control. Secondly, improved access to healthcare for this population through Health Insurance Schemes will also help in reducing their risk of CKD.

Our study also shows the feasibility of screening in resource-constrained settings and in the “hard to reach” populations. It also underscores the importance and benefits local screening programmes, without which most individuals remain ignorant of their health risks. In view of the adverse impact of renal disease on cardiovascular disease outcomes and vice versa, opportunistic screening programmes complemented by sustainable management of risk factors should be implemented in addition to strengthening the health-care workforce and making drugs affordable and accessible to local populations.

## Data Availability

The datasets used and/or analysed during the current study are available from the corresponding author on reasonable request.

## Abbreviations

ACEI: Angiotensin converting enzyme inhibitors
ARB: Angiotensin receptor blockers
BMI: Body mass index
BP: Blood pressure
CKD: Chronic kidney disease
CKD-EPI: Chronic Kidney Disease Epidemiology Collaboration
CVD: Cardiovascular disease
DBP: Diastolic blood pressure
eGFR: Estimated Glomerular Filtration Rate
ESRD: End stage renal disease
FBG: Fasting blood glucose
HDL-c: High density lipoprotein cholesterol
LDL-c: Low density lipoprotein cholesterol
MDRD: Modification of diet in renal diseases
SBP: Systolic blood pressure
Tc: Total Cholesterol
T_G_: Triglyceride
UACR: Urinary albumin excretion ratio
WC: Waist circumference
